# N6-benzyladenine and kinetin influence antioxidative stress parameters in human skin fibroblasts

**DOI:** 10.1007/s11010-015-2642-5

**Published:** 2016-01-06

**Authors:** Agata Jabłońska-Trypuć, Marzena Matejczyk, Romuald Czerpak

**Affiliations:** Department of Sanitary Biology and Biotechnology, Faculty of Civil Engineering and Environmental Engineering, Bialystok University of Technology, Białystok, Poland; The School of Medical Science in Białystok, Białystok, Poland

**Keywords:** Cytokinins, Kinetin, N6-benzyladenine, Glutathione peroxidase, Glutathione reductase, Catalase, Lipid peroxidation, Glutathione, Thiol group

## Abstract

N6-benzyladenine and kinetin are adenine-type cytokinins that play various roles in many aspects of plant development and stimulate anabolic processes in plant cells. The aim of this study was to examine the effect of N6-benzyladenine and kinetin on basic oxidative stress parameters, such as antioxidative enzyme activity, reduced glutathione and thiol group content, and lipid peroxidation. The results show a stimulatory effect of kinetin and N6-benzyladenine on antioxidative enzyme activity, as well as reduced glutathione and thiol group content. Cytokinins caused a decrease in membrane phospholipid peroxidation and exhibited protective properties against malondialdehyde production. The present findings reveal that both N6-benzyladenine and kinetin exhibit multiple and complex actions in fibroblast cells in vitro. Both show antioxidant properties and are potentially powerful agents with applications in the prevention and treatment of many diseases connected with oxidative stress in skin, for example, psoriasis.

## Introduction

According to their chemical structure, kinetin and N6-benzyladenine are adenine-type cytokinins. Cytokinins are plant hormones identified during the 1950s as substances that induce plant cell division. Numerous biochemical, physiological, and genetic studies have focused on the elucidation of the diverse functions of cytokinins in plant growth and development. Cytokinins play various roles in many aspects of plant development, including apical dominance, nutrient mobilization, root growth, senescence, and stress responses [1]. These effects appear to be the result of interactions with other plant hormones and environmental signals. Although the effects of cytokinins in plants are well known, the mechanism of their action remains to be elucidated. The group of N6-substituted adenines exhibits properties which allow them to be used in molecular medicine [2].

**Fig. 1:**
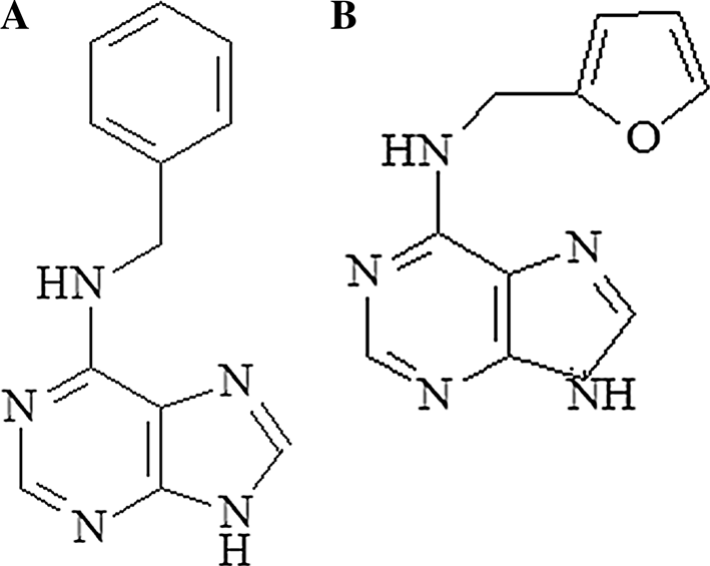
Structure of **a** N6-benzyladenine and **b** kinetin

Generally, natural cytokinins are N6-substituted adenine derivatives (Fig. 1). The first cytokinin to be identified was kinetin (K, N6-furfuryladenine), which was isolated and identified in 1955 as a degradation product of DNA that promotes cell division in plants. Kinetin is believed to be an artefactual cytokinin that originates from the autoclaving of herring-sperm DNA or forms on DNA storage over a long period of time [3, 4]. Kinetin is present in commercially available calf thymus DNA, in freshly extracted DNA from human cells in culture, in plant cell extract, and in human urine [5, 6]. The discovery of kinetin (so far recognized only as a synthetic compound) in DNA and cell extracts raised obvious question concerning the mechanism of its formation at the nucleic acid level. Evidence for the presence of kinetin in natural products has been provided by mass spectrometric analysis of DNA components [7].

The chemical structure of kinetin clearly indicates that the reaction of an adenine residue of DNA with furfural may be the possible synthetic pathway. Furfural is a dietary mutagen that is found in various food products, beverages, coffee, and white bread, and is formed when sugars are heated. It is a known constituent of the vapor phase of cigarette smoke, where it is present in large quantities (45–110 mg per cigarette). This compound has also been found in normal human plasma, urine, and heart homogenate [8]. Recent experiments show that furfural, which is the oxidized sugar residue, is formed during oxidative damage to DNA in vitro. Furfural originates from hydroxyl radical oxidation of the deoxyribose residue at the carbon 5′ and reacts with the amino group of adenine, consequently forming the Schiff base. Another intramolecular rearrangement yields kinetin in vivo [8].

These findings indicate that kinetin is an important component of a new salvage pathway of hydroxyl radicals constituting a ‘free radical sink,’ which is in compliance with the hypothesis that cytokinins are products of the oxidative metabolism of the cell. This is how cells neutralize the harmful properties of reactive oxygen species (ROS) and respond to oxidative stress by inducing the molecular mechanisms of defense and repair [1].

Recent findings suggest that cytokinins, which are known as plant-specific hormones, exhibit pleiotropic effects on human and mammalian cells. Among the cytokinins is N6-benzyladenine. N6-benzyladenine and its derivatives which regulate many cellular processes, including cell division in mammalian cells, are crucial compounds in the regulation of both the G1/S and G2/M transitions of the cell cycle, and influence the expression of the CYCD3 gene, which encodes D-type cyclin [9]. D-cyclins play a key role in regulating the passage through the cell cycle restriction point in G1 and are regulated by a variety of growth factors in animal cells. Natural aromatic cytokinins were found to inhibit several human protein kinases in non-specific manner. These kinases include conserved regulators of the eukaryotic cell cycle, CDKs. Among the CDKs, different family members control specific phases of the cell cycle. It was found that N6-benzyladenine and its derivatives led to a strong and specific inhibition of several protein kinases, such as CDK1/cyclin B, CDK2/cyclin A, CDK2/cyclin E, brain CDK5/p35, and ERK1/MAP kinase. These compounds have a strong ability to arrest cells at specific points of the cell cycle, and are particularly potent in cancer cell lines [10]. When the effect of various natural cytokinins, including N6-benzyladenine, was examined, it was found that both cytokinins-free bases and ribosides induce differentiation in leukemia cells, with similar differentiation-inducing activity in human leukemia cell lines and in plants. Cytokinin nucleosides induce mitochondrial disruption and apoptosis in cancer cells. For the manifestation of benzylaminopurine riboside (BARP) cytotoxicity, its intracellular phosphorylation is necessary. It has been suggested that caspases may be implicated in this apoptotic process [11]. Another key property of N6-benzyladenine derivatives is that they inhibit cyclic nucleotide phosphodiesterases (PDEs) in cell homogenates. PDEs regulate intracellular cAMP concentration in inflammatory cells, which is why PDE inhibition appears to be a rational goal for treating acute or chronic inflammatory diseases [12]. In the intact cell, N6-adenine derivatives do not elevate cAMP levels, thus their actions appear to be independent of the intracellular level of cAMP. These derivatives also induce cell elongation in cultures fibroblasts, decrease cell motility, and increase cell adhesion to the substratum [13, unpublished data].

Due to exposure to environmental factors, skin tissue possesses a very efficient antioxidative defense system. The most important antioxidative enzymes include glutathione peroxidase (GPX), glutathione reductase (GR), catalase (CAT), and superoxide dismutase. According to the literature, CAT and GPX are more efficient in their antioxidative protection of fibroblasts from oxidative stress than superoxide dismutase. Superoxide dismutase overexpression causes higher sensitivity to stress in fibroblasts, rather than protecting against this type of stress. CAT catalyzes the reaction of hydrogen peroxide decomposition to water and oxygen, and is a very effective enzyme, causing the decomposition of 6 million water particles in 1 min. It occurs in the peroxisomes, mitochondria, endoplasmatic reticulum, and cytoplasm in plant and animal cells. CAT activity in skin fibroblasts significantly decreases along with senescence processes [14].

Other element of the antioxidative defense system are the GPXs, which have an ability to reduce non-organic and organic peroxides. The activity of peroxidases in skin fibroblasts increases along with the senescence, which has a protective effect on membrane phospholipids through the inhibition of their peroxidation. The other enzyme associated with glutathione metabolism is GR, which occurs in the mitochondria and cytoplasm of cells, and as a reductive factor, uses NADPH. GR maintains appropriate concentration of GSH (reduced glutathione).

There is a lack of data concerning the effect of N6-benzyladenine and kinetin on normal fibroblast metabolism, which is why we chose to examine selected parameters under physiological conditions without any stress factors, with the aim of showing the effect of selected cytokinins on human skin. Therefore, our study focused on the most appropriate research model: fibroblasts, which account for the largest number of cells in skin. The aim of this study was to examine the effect of N6-benzyladenine and kinetin on basic oxidative stress parameters, such as antioxidative enzyme activity, reduced glutathione and thiol group content, and lipid peroxidation. We also wanted to determine whether the compounds stimulated oxidative stress in fibroblasts or whether they prevented free radical synthesis. We also attempted, for the first time, to correlate the relationship between N6-benzyladenine and kinetin molecular concentration, and the appropriate cellular effects.

## Materials and methods

### Chemicals and reagents

Kinetin, N6-benzyladenine, phosphate-buffered saline (PBS), DMEM, FBS, 5,5′-dithiobis(2-nitrobenzoic acid) (Ellman’s reagent), hydrogen peroxide, NADPH, meta-phosphoric acid (MPA), SDS, TCA, TBA, and the GPX Cellular Assay Kit were purchased from Sigma (St. Louis, MO, USA). All other reagents and solvents were of analytical grade. Tissue culture dishes and flasks were purchased from Sarstedt.

### Cell culture

The effect of kinetin and N6-benzyladenine was examined in normal human skin fibroblasts. The fibroblast cell line was derived from 10 patients between the ages of 30 and 40 years, and was maintained in DMEM supplemented with 10 % FBS at 37 °C in a humified atmosphere of 5 % CO_2_ in air. Adherent cells (1 × 10^5^ cells/ml) in 2 ml of culture medium were incubated with or without the test compounds in tissue culture 6-well plates. The cell number was counted at N6-benzyladenine and kinetin concentration of 10^−4^, 10^−5^, 10^−6^, and 10^−7^ M. Antioxidant enzyme activity, reduced glutathione and SH group content and lipid peroxidation, was examined at kinetin concentrations of 10^−5^ and 10^−6^ M and N6-benzyladenine concentrations of 10^−6^ and 10^−7^ M.

### Chemical treatment of cells

Kinetin and N6-benzyladenine were stored in a freezer at temperature under −4 °C, protected from light. The cytokinins were added to the cultured cells for a final concentration in the range of 10^−5^ to 10^−6^ M for kinetin and 10^−6^ to 10^−7^ M for N6-benzyladenine. The control cells were incubated without the test compounds.

### Estimation of cell number

The number of fibroblasts was determined by direct counts of cells in the growth medium using a Bürker chamber.

In all the assays, data were normalized to each other by measuring the total cell number to exclude the risk that variable cell numbers were lysed.

### Enzyme assays

For enzyme analysis, cells were rinsed with PBS at 4 °C and collected by scraping in cold PBS, centrifuged, and resuspended in 1 ml of PBS and stored at −80 °C. Cells were lysed by freezing and thawing to room temperature twice. Aliquots of the cell lysates were collected for enzyme assays. GPX (EC 1.11.1.9) activity was measured according to the method of Paglia and Valentine, using the GPX Cellular Activity Assay kit (Sigma Aldrich) [15]. An indirect determination method is based on the oxidation of glutathione (GSH) to oxidized glutathione (GSSG) catalyzed by GPX, which is then coupled to the recycling of GSSG back to GSH utilizing GR and NADPH. The decrease in NADPH absorbance measured at 340 nm during the oxidation of NADPH to NADP^+^ was indicative of GPX activity, since GPX is the rate limiting factor of the coupled reactions. CAT (EC 1.11.1.6) activity was measured spectrophotometrically at 240 nm by monitoring the decline in H_2_O_2_ in the presence of cellular lysates [16]. Activity was calculated using the rate of change per min and the molar extinction coefficient (*λ*_240_ = 47) of H_2_O_2_. GR (EC 1.8.1.7) activity was determined by the method reported by Mize and Langdon [17]. The reaction mixture consists of 1 ml 0.25 M KCl in 0.02 M phosphate buffer (pH 7.0), 0.25 ml 7 mM GSSG, 0.3 ml aliquots of the cell lysates, and 0.5 ml 0.7 mM NADPH. The consumption of NADPH was monitored spectrophotometrically at 340 nm. Enzyme activity was calculated using the molar extinction coefficient of 6.22 mM/cm.

### Glutathione assay

Cells collected for glutathione determination were rinsed with PBS at 4 °C and scraped from Petri dishes (between 1 and 5 × 10^6^/plate). The cells were then resuspended in 1 ml of cold PBS and lysed by freezing and thawing to room temperature twice. Subsequently, 1 ml of MPA working solution was added. Cells were incubated at 4 °C for 1 h and centrifuged at 13,500×*g* for 10 min. The upper clear aqueous layer was used for the assay. Reduced GSH was determined using the Glutathione Assay kit (Merck). In this assay, chromophoric thione was obtained with a maximal absorbance at 400 nm.

### Determination of SH groups

For the determination of total content of SH groups in fibroblasts, cells were washed twice with PBS (pH 7.4) at 4 °C and dispersed by scraping. The cells were counted, resuspended in 1 ml of PBS, and collected by centrifugation at 5000×*g* for 10 min. The cell pellet was resuspended in 1 ml of 0.5 M phosphate buffer (pH 7.8) containing 0.1 % SDS. Then, 25 µl Ellman’s reagent (5 mM) was added and the thiol groups were measured spectrophotometrically at 412 nm using the molar extinction coefficient of 13.6 mM/cm.

### Determination of TBA reactive species (TBARS) levels

The level of membrane lipid peroxidation products, or TBARS, was measured using the method of Rice-Evans et al. [18]. The cells were washed with PBS (pH7.4), scraped from Petri dishes and resuspended in 1 ml of PBS (between 1 and 5 × 10^6^ per plate). TCA (15 %, 1 ml) and TBA (0.37 %, 1 ml) were added to 1 ml of the cell suspension and mixed. This mixture was submerged in a boiling water bath for 10 min and the concentration of TBARS was assessed spectrophotometrically at 532 nm using the molecular extinction coefficient of 156 mM/cm.

### Statistical analysis

A minitab statistical package was used to carry out one-way ANOVA. The Student’s *t* test was used to estimate differences between means at 5 % level of significance.

## Results

### Cell number

**Fig. 2:**
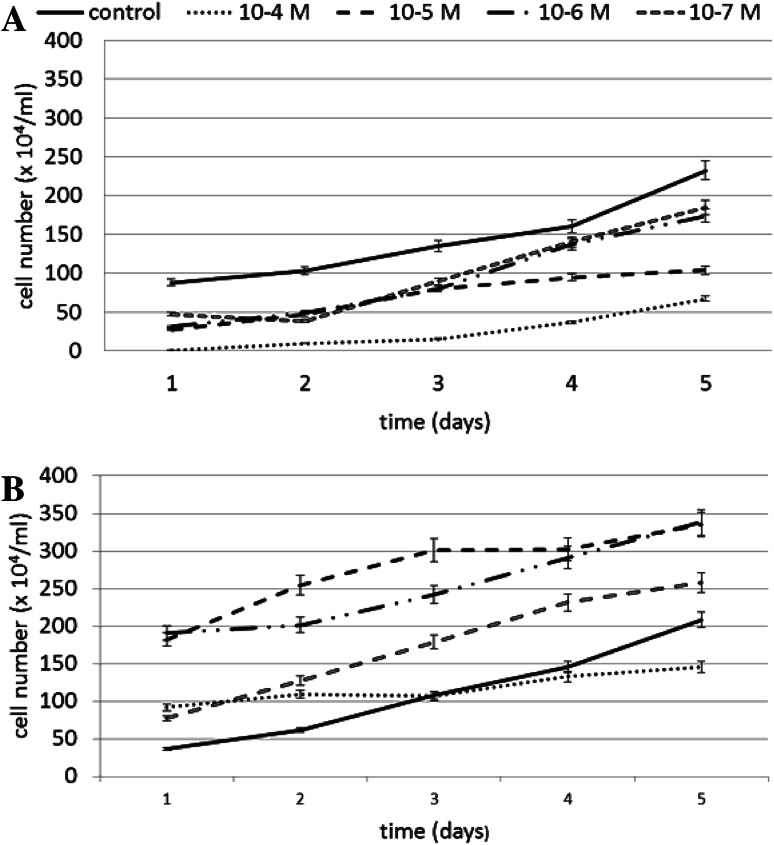
The effect of various concentrations of **a** N6-benzyladenine and **b** kinetin on fibroblast cell number during a 5-day incubation (SE < 5 %)

N6-benzyladenine caused a significant decrease in cell number. The slightest effect was observed on day 4 at N6-benzyladenine concentrations of 10^−6^ and 10^−7^ M, resulting in a decrease of 14 and 12 %, respectively, compared to the untreated control cells. The decrease in cell number was proportionate to the increase in N6-benzyladenine concentration, and it was lowest at 10^−4^ M N6-benzyladenine. N6-benzyladenine did not inhibit cell division completely, and mitotic divisions were observed. However, the number of cells was significantly decreased compared to the control. The effect of N6-benzyladenine on cell number is shown in Fig. 2a.

Kinetin stimulates cell division in cultured cells. The optimal concentrations of kinetin were determined to be 10^−5^ and 10^−6^ M at the two concentrations, the total cell number was significantly higher than in the control. The number of living cells was particularly increased at 10^−5^ and 10^−6^ M kinetin on days 1 and 2. At 10^−4^ M, a decrease compared to the control was observed, particularly on days 3 and 4. The effect of kinetin on cell number is shown in Fig. 2b.

We therefore selected the two concentrations of N6-benzyladenine and the two concentrations of kinetin at which the total cell number was the highest among the cultures, and then tested the oxidative stress parameters at each of the two concentrations.

### Antioxidative enzyme activity

**Fig. 3:**
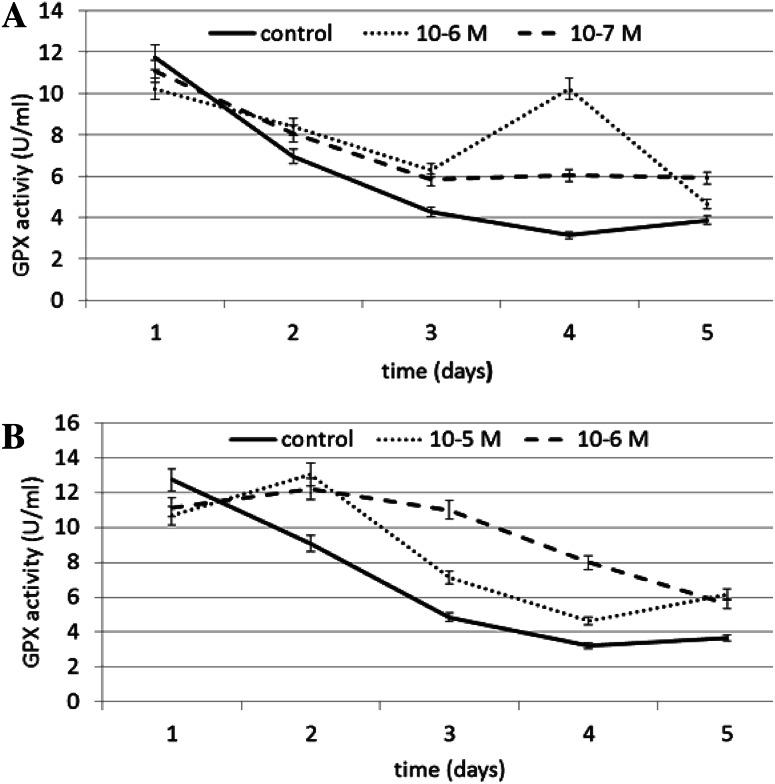
The effect of selected concentrations of **a** N6-benzyladenine and **b** kinetin on GPX activity during a 5-day incubation (SE < 5 %)

The effect of N6-benzyladenine on GPX (EC 1.11.1.9) activity is shown in Fig. 3a. A significant increase in GPX activity of ~225 % compared to the control was observed in N6-benzyladenine-treated cells at concentration of 10^−6^ M on day 4. N6-benzyladenine at a concentration of 10^−7^ M also increased GPX activity by 92 % compared to the control. On day 1, a slight decrease in GPX activity compared to the control was observed (12 % at 10^−6^ M and 5 % at 10^−7^ M). A N6-benzyladenine concentration of 10^−6^ was most efficient at stimulating GPX activity.

The effect of kinetin on GPX activity is shown in Fig. 3b. Kinetin significantly increased GPX activity on days 3, 4, and 5. At 10^−6^ M on day 4, kinetin caused an increase in GPX activity of ~147 % compared to the control. On day 5, 10^−5^ M kinetin caused an increase in GPX activity of ~68 % compared to the control. On day 3, 10^−6^ M kinetin also stimulated enzyme activity, increasing it by ~127 % compared to the control.

**Fig. 4:**
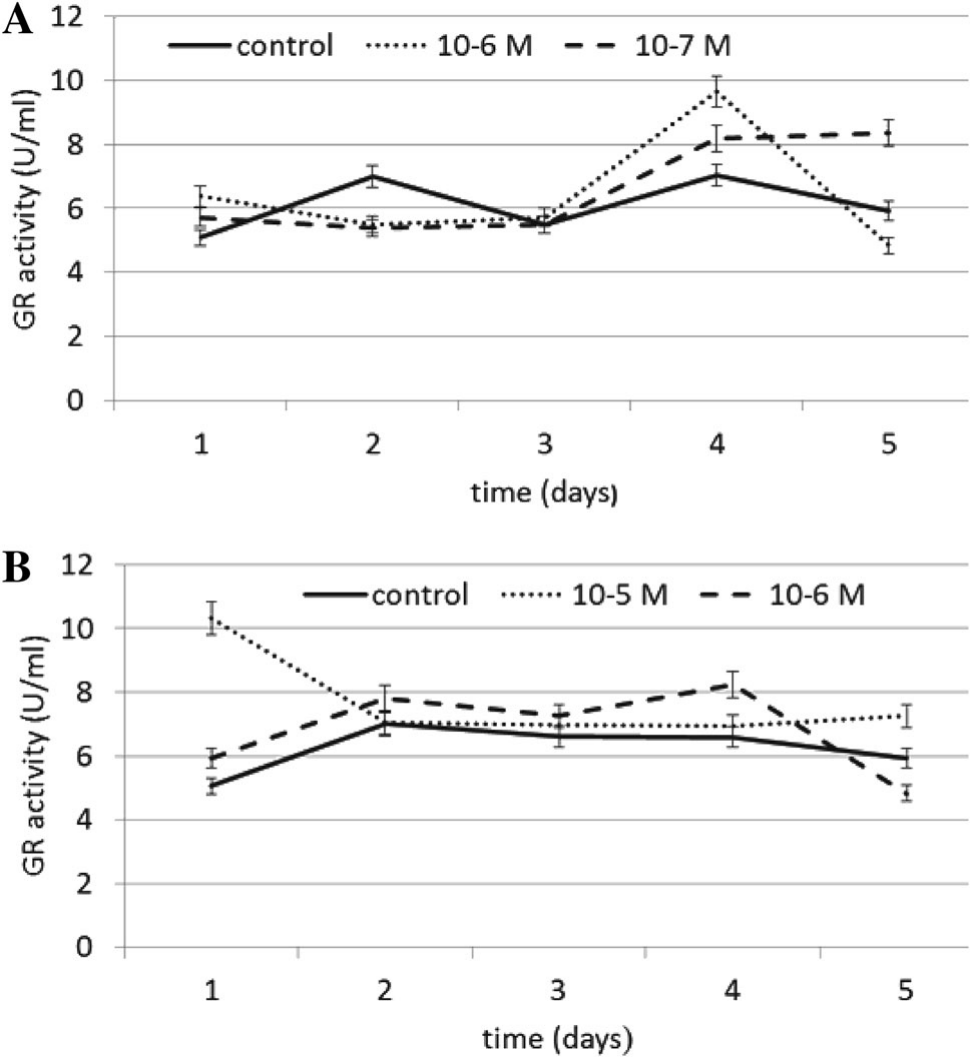
The effect of selected concentrations of **a** N6-benzyladenine and **b** kinetin on GR activity during a 5-day incubation (SE < 5 %)

The effect of N6-benzyladenine on GR (EC 1.8.1.7) activity is shown in Fig. 4a. Similarly to GPX, a significant increase in GR activity of ~36 % compared to the control was observed on day 4 at both the concentrations tested. A slight decrease in enzyme activity was observed on day 2. A N6-benzyladenine concentration of 10^−6^ M was also most efficient at stimulating GR activity.

The effect of kinetin on GR activity is shown in Fig. 4b. A significant increase in GR activity of ~104 % compared to the control was observed at a concentration of 10^−5^ M kinetin on day 1. The concentration of 10^−6^ M kinetin resulted in a less significant increase, of ~24 % compared to the control, observed on day 4.

**Fig. 5:**
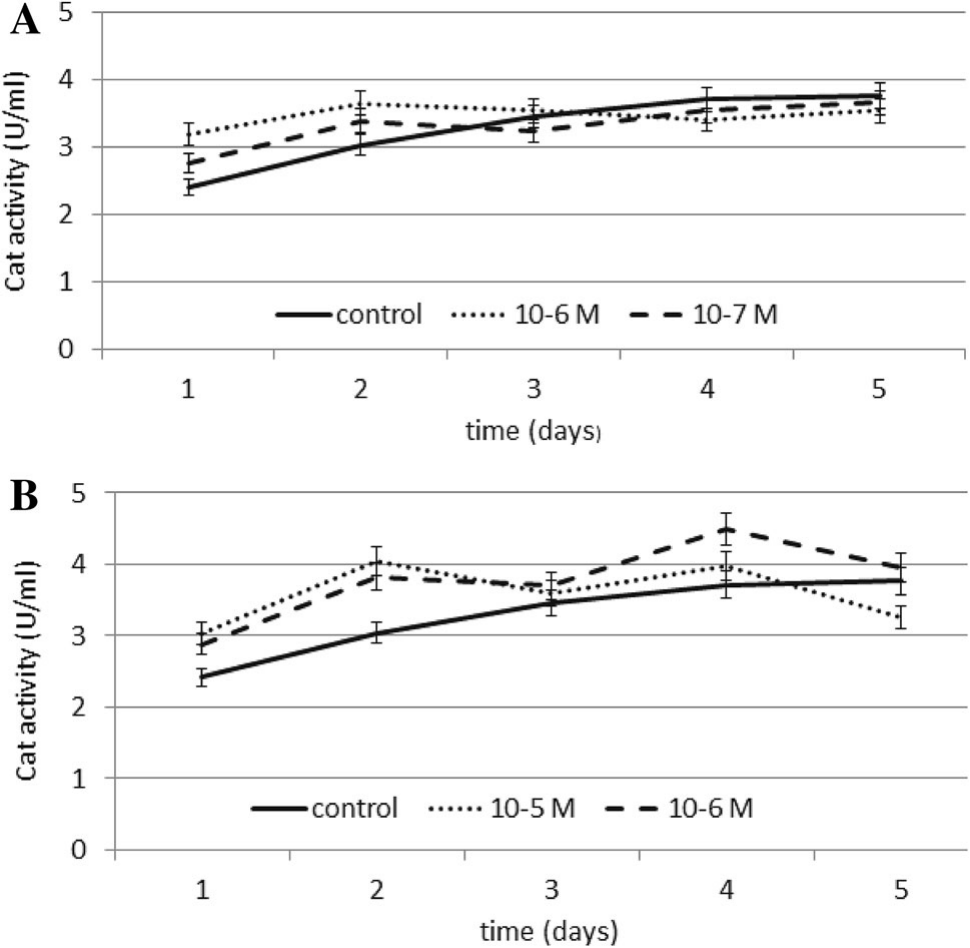
The effect of selected concentrations of **a** N6-benzyladenine and **b** kinetin on Cat activity during a 5-day incubation (SE < 5 %)

The effect of N6-benzyladenine on CAT (EC 1.11.1.6) activity is shown in Fig. 5a. Compared to the control, N6-benzyladenine increased CAT activity on days 1 and 2 by ~32 % at 10^−6^ M and by ~14 % at 10^−7^ M on days 3, 4, and 5; a slight decrease in CAT activity was observed at both the concentrations tested compared to control.

The effect of kinetin on CAT activity is shown in Fig. 5b. The most significant stimulation of CAT activity in kinetin-treated cells was observed on day 2 at both the concentrations tested: at 10^−5^ M, kinetin caused an increase of ~33 % in enzyme activity compared to the control, while at 10^−6^ M it was less effective, resulting in an increase of 25 %. At 10^−5^ M kinetin on day 5, there was a slight decrease in CAT activity compared to the control.

The results show a stimulatory effect of kinetin and N6-benzyladenine on antioxidative enzyme activity.

### Reduced glutathione content

**Fig. 6:**
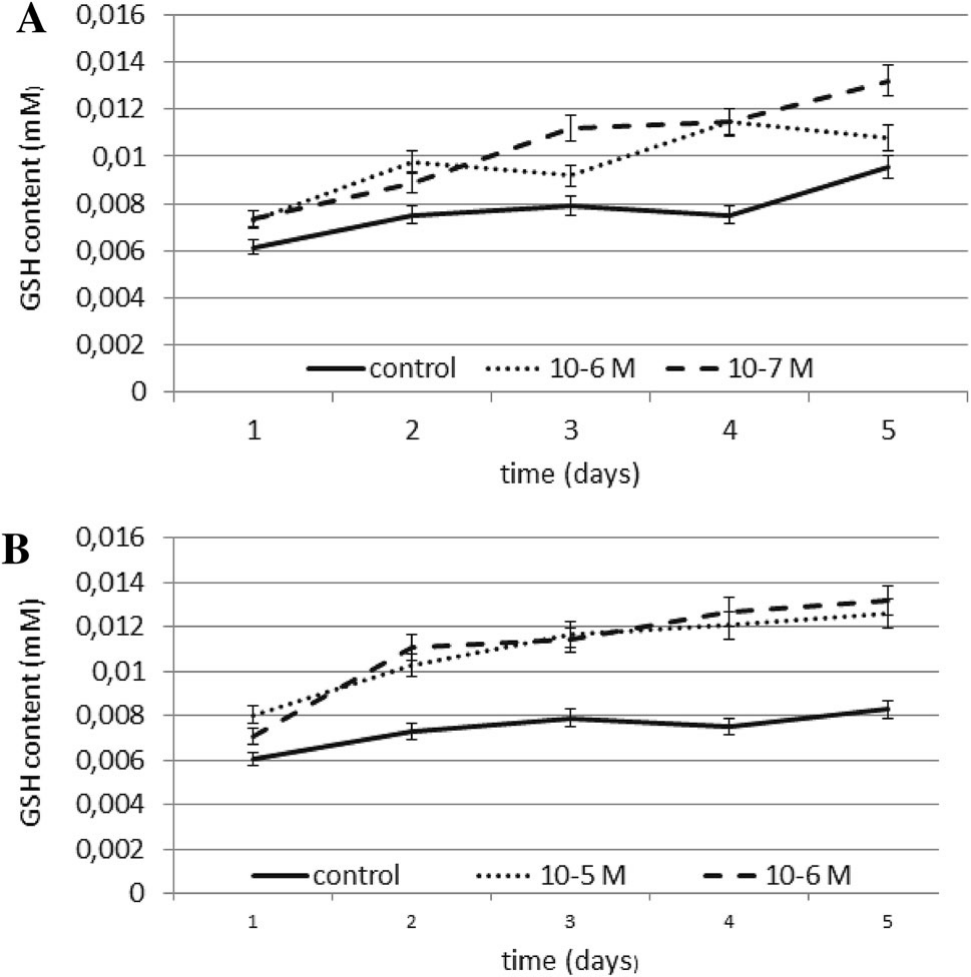
The effect of selected concentrations of **a** N6-benzyladenine and **b** kinetin on GSH content during a 5-day incubation (SE < 5 %)

N6-benzyladenine at both the concentrations tested caused increase of ~50 % compared to the control. No decrease below control levels was observed. Kinetin caused an increase of ~60 % at a concentration of 10^−5^ M compared to the control, and of ~68 % at 10^−6^ M compared to the control. Reduced glutathione content was relatively high at both the kinetin and N6-benzyladenine concentrations tested. This indicates a stimulatory effect of kinetin and N6-benzyladenine on GSH biosynthesis in fibroblasts. The effect of N6-benzyladenine on GSH content is shown in Fig. 6a, and the effect of kinetin on GSH content is shown in Fig. 6b.

### SH group content

**Fig. 7:**
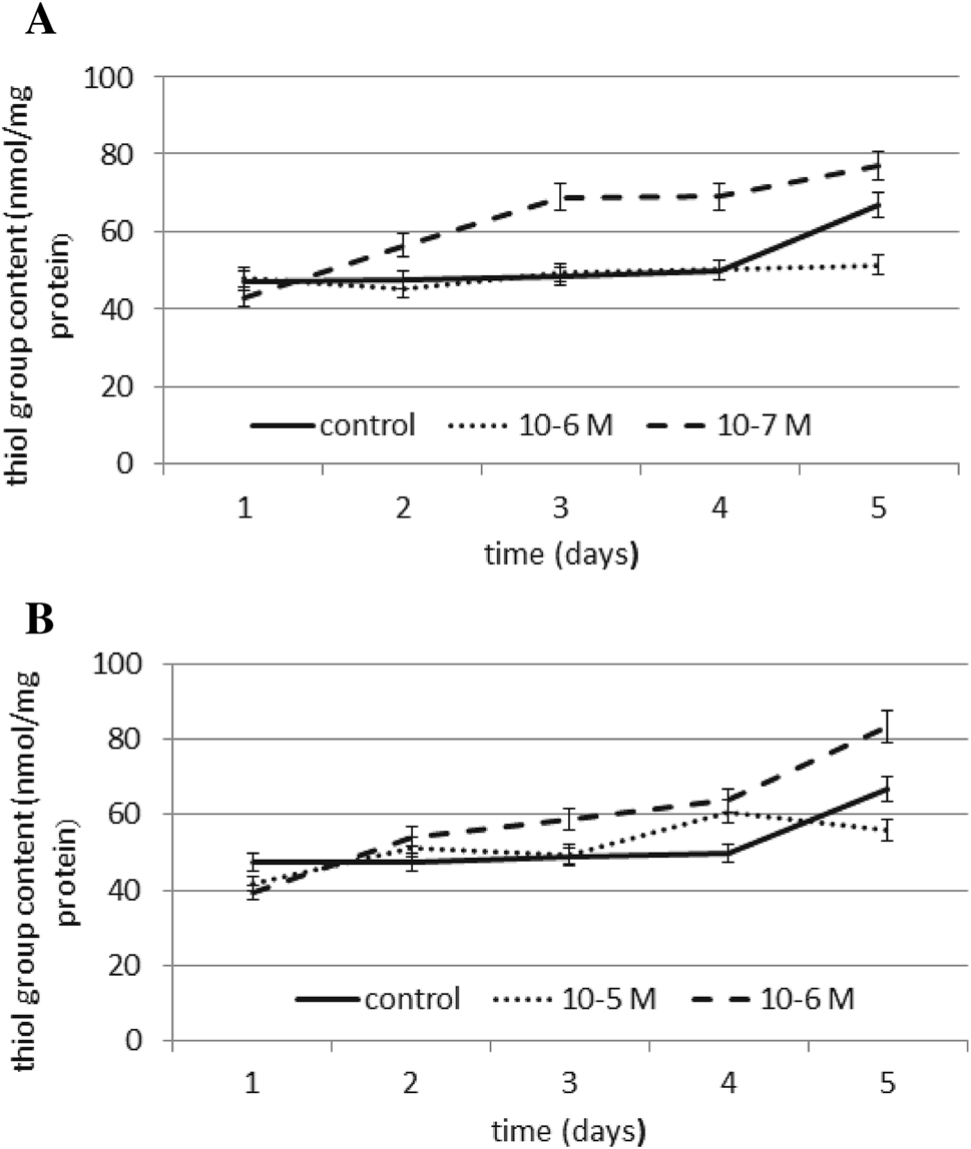
The effect of selected concentrations of **a** N6-benzyladenine and **b** kinetin on SH group content during a 5-day incubation (SE < 5 %)

The effect of N6-benzyladenine on SH group content is shown in Fig. 7a, and the effect of kinetin on SH group content is shown in Fig. 7b. To determine the oxidation of the SH group, a spectrophotometric assay with Ellman’s reagent was used. Thiol group content was evaluated as a marker of protein oxidation. A significant increase in thiol group content of ~40 % compared to the control was observed on days 3 and 4 at a concentration of 10^−7^ M N6-benzyladenine. Exposure to N6-benzyladenine resulted in ~9 % decrease in the total cellular content of the thiol groups only on day 1. At both the concentrations of kinetin tested, a significant increase of ~25 % in thiol group content compared to the control was observed on day 4. Exposure to kinetin resulted in a ~15 % decrease in the total cellular content of the thiol groups only on day 1. These data indicate that the tested cytokinins do not cause oxidative damage in proteins.

### Lipid peroxidation

**Fig. 8:**
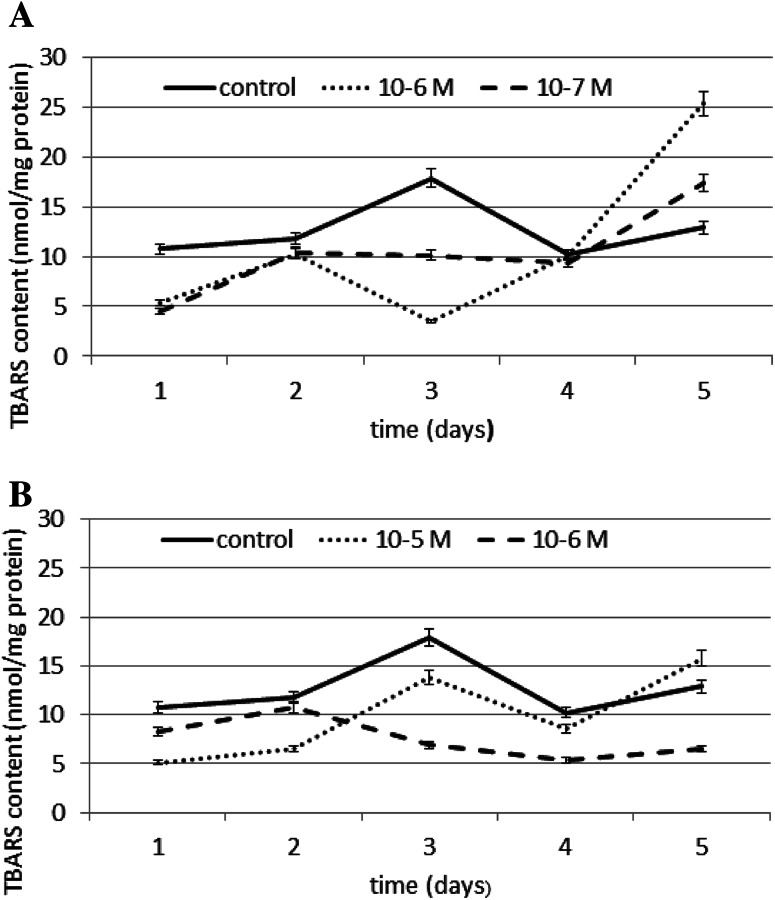
The effect of selected concentrations of **a** N6-benzyladenine and **b** kinetin on TBARS content during a 5-day incubation (SE < 5 %)

The effect of N6-benzyladenine on lipid peroxidation is shown in Fig. 8a, and the effect of kinetin on lipid peroxidation is shown in Fig. 8b. TBARS content was measured as an index of lipid peroxidation. The results showed a significant difference between TBARS levels in the control, kinetin, and N6-benzyladenine-treated cells. The addition of these cytokinins to the cells induced a significant reduction in TBARS content compared to the control. At a N6-benzyladenine concentration of 10^−6^ M, a significant decrease in TBARS content of ~80 % compared to the control was observed on day 3. However, N6-benzyladenine was most effective at a concentration of 10^−7^ M, resulting in a decrease of 58 % compared to the control on day 1. Kinetin at a concentration of 10^−5^ M induced a decrease in TBARS content of ~50 % compared to the control, observed on day 1 and 2. However, kinetin was most effective at a concentration of 10^−6^ M, resulting in a decrease of 61 % compared to the control on day 3. The obtained results suggest that the cytokinin demonstrates protective properties against TBARS production and as a consequence, decreases membrane phospholipid peroxidation.

## Discussion

Cytokinins are one of the groups of phytohormones, and their effect on metabolism in plants is quite well-known. However, there is a lack of information concerning their effect on human or animals cells and organisms. Fibroblast cell cultures are a very appropriate experimental model for researching the effect of cytokinins on selected parameters associated with oxidative stress under physiological conditions. The present study demonstrated the biological activity of selected cytokinins in cultured human skin fibroblasts derived from donors aged 30 to 40 years. To evaluate the optimal cytokinins concentrations, we investigated their effect on cell number in a broad spectrum of concentrations (dose–response curve), and selected the two most effective concentrations for each compound. Our aim was to study the antioxidative properties of kinetin and N6-benzyladenine; therefore, we investigated antioxidative enzyme activity, reduced glutathione and thiol group content, and the levels of lipid peroxidation products under treatment with selected concentrations of the cytokinins.

Since the skin donors were between the ages 30–40 years, the favorable action of phytohormones on skin cells may not only have therapeutic potential, but may also play a role in the delay of senescence. Aging of the skin and human organism may be both a result and a cause of the very high biosynthesis of ROS. Cumulative oxidative damage as an important factor in aging is supported by a large body of experimental findings [19, 20]. ROS cause damage to DNA, proteins and lipids, and are one of the major factors affecting cellular senescence. Skin is a tissue exposed to environmental agents, such as ultraviolet radiation and ozone, and therefore possesses extremely efficient antioxidant activity. The reduction in efficiency of this system has been proposed as a factor in skin aging.

ROS generation is observed in many diseases, in aging, and also under normal conditions in skin cells. For this reason, we focused our research on the basic parameters of oxidative stress in fibroblasts under normal conditions, without the presence of any stress producing compound, such as hydrogen peroxide or UV light. In general, the free radical theory of aging states that, during the aging process, there is an accumulation of oxidative products, such as oxidized proteins, DNA adducts, and lipid metabolites. In addition, there is a significant decrease in the antioxidant defense system. Since it is a highly metabolic tissue with the largest surface area in the body, skin is a major target of oxidative stress. Skin tissue is exposed to a variety of damaging species which originate from exogenous and endogenous sources. This is why skin is equipped with defense mechanisms against oxidative stress, such as enhanced physical stability against oxidative damage, prevention of the production of ROS from endogenous sources, repair mechanisms, and the antioxidant defense system. This defense system is composed of antioxidant enzymes and low molecular weight antioxidants (glutathione) [21].

Our results showed that kinetin significantly stimulated fibroblasts proliferation, causing a considerable increase in cell number, in particular at concentrations of 10^−5^ and 10^−6^ M, while N6-benzyladenine caused a decrease in the total cell number.

In the plant model, Riou-Khamlichi et al. [9] have shown that kinetin activates cellular division via the induction of cyclin D—CycD3 in *Arabidopsis thaliana*. Therefore it seems likely that this hormone can act similarly in human and animal cells, by the activation of cyclin-dependent kinases, and thus result in stimulation of cell proliferation. Kinetin also affects the MAP kinases pathway, which can influence cell proliferation, and thus, in this way, the hormone may affect the mitotic division of fibroblasts [7]. According to the literature, kinetin can cause an increase in total DNA amount in fibroblast cell nucleus [22]. In the S phase of the cell cycle in the cell nucleus, an increase in DNA amount is observed. It occurs due to intensive biosynthesis of nucleic acids. One of the markers of cellular aging is the arrest in the G1 phase and the impossibility of the transition to the S phase, which cannot be reversed by the activity of any physiological mitogens. The reason for the cell’s entry into this cell cycle stage may be selective repression of certain genes encoding growth, the expression of which is necessary for exit from the G1 phase and the start of DNA synthesis [19]. Therefore, kinetin may be a factor that causes an increase in DNA content in the nucleus of fibroblasts and it will stimulate the cells to re-enter the cell cycle. It is possible that kinetin unlocks repression of specific genes, allowing them to activate and then the cells can re-enter the path of mitotic divisions. Our study showed particularly strong stimulating effect on cell proliferation by kinetin in the first days of the experiment, that is, after 24 h after hormone administration on cell culture.

N6-benzyladenine is a compound which has less impact on the proliferative capacity of cells than kinetin, and it even inhibits their proliferation, which is in consistent with literature reports on the effects of this compound. N6-benzyladenine and its derivatives affect the cell cycle stopping it at certain stages by enhancing expression of a gene CYCD3, which encodes the essential cell cycle proteins—cyclin. Benzyladenine analogs by inhibiting CDK also slowed cell proliferation activity. These tests were performed on tumor cells, especially myeloid leukemia cells [23, 24]. Own research also confirms that benzyladenine inhibits cell proliferation. At concentrations 10^−6^ and 10^−7^ M, it acts relatively least on halting division in the fibroblasts and therefore these concentrations were used for further studies. Probably, in fibroblasts benzyladenine uses similar mechanisms to inhibit proliferation in tumor cells, i.e., it causes the appearance of a strong and specific inhibition of important protein kinases CDK. These factors play an important role in both mitosis and in the early stages of cell division (interphase).

By contrast, the two tested compounds caused an increase in antioxidative enzyme activity. In every case, increased activity in stimulated cells compared to untreated control cells was observed. Our results are in compliance with literature; according to Sharma et al. [25, 26], kinetin acts as an antioxidant because it stimulates antioxidative enzyme activity and prevents oxidative stress, causing, for example, an increase in CAT activity. Studies in our laboratory have revealed that both kinetin and N6-benzyladenine activate GPX and GR and maintain constant GSH content in fibroblasts. According to the literature [7], the antioxidative activity of kinetin results from its ability to create complexes with copper ions, which have an activity analogous to superoxide dismutase. This compound acts as a ROS scavenger, as oxygen radicals directly abstract hydrogen from the α-carbon of the amine bond of kinetin or undergo a faster dismutation reaction in aqueous solution, when kinetin is complexed with copper. Our results also indicated that N6-benzyladenine has distinct antioxidative properties, as well. According to the literature [27], N6-benzyladenine as a complex with cupric ion (II) has superoxide dismutase mimetic activity. We demonstrated that N6-benzyladenine activates antioxidative enzyme activity, in particular GPX and GR. N6-benzyladenine, like other biologically active chemical compounds originating from plants, may act through many different mechanisms. Among the mechanisms proposed to explain the biological activity of N6-benzyladenine is its capacity to bind protein and eventually influence enzyme activity by either competitive or allosteric interactions: regulation of signal transduction; modulation of redox-sensitive transcription factors, including Nrf2, NF-κB, and AP-1; glutathione biosynthesis and gene expression in general. N6-benzyladenine binds a specific receptor, activates the kinase cascade, and, as a consequence, causes specific gene activation.

According to the literature [28], MAP kinases are involved in the regulation of CAT activity. The best-known members of the mitogen-activated protein kinase (MAPK) family are p42/44 MAPK (also known as external signal regulated kinase, ERK), p38MAPK and Jun terminal kinase (JNK). ERK is mainly involved in the regulation of growth and proliferation. They are also activated by mitogenic stimuli: JNK and p38MAPK, however are activated by different stresses. It is likely that the up-regulation of CAT expression by N6-benzyladenine is controlled by regulatory mechanisms acting on CAT mRNA stability through activated p38MAPK. The activation of p38MAPK is often associated with enhanced mRNA stability of a number of stress-related genes [28]. On the other hand, Zhou et al. [29] indicated that NF-κB is associated with the transcriptional regulation of CAT. Similar mechanisms may activate GPX and GR. There is no evidence that N6-benzyladenine influences antioxidative enzyme activity. It is possible that the activity of this cytokinin is similar to that of flavonoids, a better-known group of biologically active substances occurring in plants. However, the molecular mechanism of flavonoids and their effect on GSH-connected enzymes is unknown.

A recent article indicated that the effects of flavonoids on antioxidant enzyme expression and activity may be mediated by the modification of signal transduction pathways [30]. Chemical compounds (e.g., N6-benzyladenine or kinetin) activate a number of cellular kinases, including MAPKs and PI3K, and both pathways are probably involved in the up-regulation of the activity of several antioxidant enzymes. Both the PI3 K/AKT and MAPK pathways have been implicated in the mechanism of activation of the transcription factor Nrf2, which acts through the antioxidant-response element to initiate gene transcription. Nrf2 is the basic leucine-zipper transcription factor, a nuclear factor-erythroid 2 p45-related factor 2, and has been shown to play a crucial role in protecting cells from oxidative stress. In certain situations, Nrf2 dissociates from its cytosolic inhibitor Keap1, translocates to the nucleus,and binds to antioxidant-response elements (AREs) in the promoters of target genes. This activity leads to the transcriptional induction of cellular defenses genes, including glutathione biosynthetic enzymes and GSH-dependent antioxidant enzymes [31]. The same or similar mechanism may explain the increased levels of GSH in cells treated with N6-benzyladenine and kinetin.

Kinetin in plant cells has been found to prevent unsaturated fatty acid oxidation in the cell membrane [32]. This is in agreement with our results: we observed a significant decrease in TBARS content in fibroblasts, possibly associated with a decrease in membrane phospholipid oxidation. In kinetin-treated fibroblasts, we observe an increase in TBARS content, which may explain an observed increase in collagen biosynthesis (unpublished data). According to the literature [33], TBARS and other lipid peroxidation products (mainly carbonyls) stimulate collagen biosynthesis in fibroblasts. The molecular mechanism behind the effect of TBARS on collagen gene expression is yet to be determined, but may be similar to those of acetaldehyde activity. Bonds between proteins and aldehydes (4-hydroxynonenal—4-HNE, TBARS, or MDA) may play an important role in the increase of the transcription of the genes responsible for collagen biosynthesis. Aldehydes theoretically create bonds with all cell components, including DNA, which is why they may stimulate collagen gene expression through direct interactions with regulatory DNA cis-elements.

Abovementioned 4HNE is one of the main end products of lipid peroxidation, which has gained a lot of attention because of its impact on many cellular processes, especially on cell growth. Growth stimulating influence effect of HNE is achieved trough modulation of various signaling pathways, e.g., interactions with basic fibroblast growth factor, activation of Nrf2/ARE pathway, or induction of c-Fos expression. Although we did not analyze 4-HNE, we suppose that its content may also change during the experiment, similar to analyzed TBARS and those changes may potentially have impact on cell growth and proliferation. According to the literature, 4-HNE as a “second messenger” of reactive oxygen species might exert growth modifying activities, what was indicated in HeLa cell line, where initial inhibition of proliferation was followed by the increase in cell growth [34, 35]. We also want to stress the connection observed in our experiment between cell proliferation and reactive aldehydes concentration. We observed changes in cell proliferation associated with changes in reactive aldehydes content. Reactive aldehydes such as HNE are involved in cellular processes not only under aberrant conditions but also in normal physiology, as in our study. It is in agreement with literature data, which indicate that HNE acts as a growth regulator not only for malignant cells but also for fibroblasts and other mesenchymal cells such as bone [36].

In contrast to kinetin, we observed a decrease in TBARS content in N6-benzyladenine-treated fibroblasts. This may indicate that N6-benzyladenine inhibits membrane phospholipids peroxidation and possibly decreases oxidative stress in these cells. Oxidative damage to important molecules in cells, such as proteins, lipids, or DNA, may be deleterious. Their oxidative modifications by ROS are implicated in the pathogenesis of both normal aging and many diseases. To assess the effect of N6-benzyladenine on protein, in this study we used one of the most widely studied markers of protein oxidation: protein thiol groups. No significant SH group oxidation was observed in N6-benzyladenine-treated cells. These effects may be attributed in part to the antioxidative activity of the examined cytokinins.

The alternative explanation for enhanced antioxidative enzyme activity and increased levels of GSH may be protein kinase A (PKA) activation. It is possible that N6-benzyladenine activates PKA via interactions with other cellular signaling pathways, such as TGF-β and endothelin-1. It is known that TGF-β activates PKA via a cAMP-independent pathway and this activation is also regulated by the smad3/4 complex. However, stimulation of PKA activity by endothelin-1 involves the degradation of IκB [37].

On the other hand, the level of intracellular cAMP under the influence of tested compounds was not yet directly measured, so it is not known whether N6-benzyladenine changes this parameter in the cell. It is also possible that benzyladenine increases cAMP level in a highly specialized, small area within the cell or works completely independent of cAMP. Benzyladenine can activate protein kinase or with cAMP affect the distribution of intracellular Ca^2 +^, Na ^+^, and K ^+^, or it can directly interact with the microtubules and microfilaments and change the shape of the cell. According to the literature, benzyladenine and its derivatives have proved to be selective inhibitors of phosphodiesterase (PDE). PDEs are a family of enzymes commonly found in mammalian tissues, which by hydrolysis of cAMP and cGMP mediate cellular signal transduction pathways. Inhibition of PDE by benzyladenine may be used for the potential treatment of diseases related to inflammation, since these enzymes play a significant role in creating inflammation. By inhibiting PDE, benzyladenine and its derivatives also inhibit the synthesis of tumor necrosis factor TNF-α, which is a key cytokine which affects the development of an inflammatory disorder. Literature data indicate that PDE inhibitors are also anti-TNF-α factors. They are used in clinical practice for the treatment of a number of diseases where inflammation is observed, e.g., asthma, atopy skin, chronic obstructive pulmonary disease, rheumatoid arthritis, and neurological diseases [12, 38, 39]. Benzyladenine, which most likely also acts by the mechanism of inhibition of phosphodiesterases appears to fulfill the conditions to act anti-inflammatory and may be used in the therapy of diseases with inflammation.

A positive effect of kinetin on proteins possessing thiol groups was also observed. Under kinetin treatment, the thiol group content was significantly higher than in the control. These results are in accordance with the relatively high GSH content. GSH thiol groups are in balance with protein thiol groups. According to Markovic et al. [40], GSH is an important factor in cell proliferation as it regulates telomerase activity in cells, and its presence is required in the nucleus during the early phases of cell division and growth. Reduced glutathione may be taken up from the cytoplasm into the nuclei either passively or through energy-dependent transport. This compound may also be synthesized de novo in the nucleus by the enzymes *γ*-glutamylcysteine synthetase and GSH synthetase. It is essential in the cell nucleus since a large number of nuclear proteins, including transcription factors, require a reduced environment to bind DNA. Approximately 60 proteins are directly involved in transcription, nucleotide metabolism, phosphorylation, and dephosphorylation or ubiquitinylation. All the processes are essential for cell cycle progression. GSH in the nucleus may act as a transcriptional regulator of NF-κB, AP-1, and p53 protein by altering their nuclear redox state [40]. It appears that kinetin may affect cell proliferation and other processes by increasing GSH content in cells.

Kinetin has a positive effect on skin, as it normalizes hyperpigmentation and improves the structure of aged skin. In addition, it has no adverse effects on skin and is safe for long-term treatment [41]. Our results indicate that both kinetin and N6-benzyladenine exhibit multiple and complex action in fibroblast cells in vitro, and act favorably on basic oxidative stress parameters, showing antioxidant properties. We suggest that both the tested compounds have antioxidative properties which allow them to be used as therapeutic agents for prevention and therapy of many skin diseases, in particular those connected with oxidative stress in skin, such as psoriasis.
